# Low extracellular magnesium does not impair glucose-stimulated insulin secretion

**DOI:** 10.1371/journal.pone.0217925

**Published:** 2019-06-04

**Authors:** Lisanne M. M. Gommers, Thomas G. Hill, Frances M. Ashcroft, Jeroen H. F. de Baaij

**Affiliations:** 1 Department of Physiology, Radboud Institute for Molecular Life Sciences, Radboud University Medical Centre, Nijmegen, The Netherlands; 2 Department of Physiology, Anatomy & Genetics, University of Oxford, Oxford, United Kingdom; Centre National de la Recherche Scientifique, FRANCE

## Abstract

There is an increasing amount of clinical evidence that hypomagnesemia (serum Mg^2+^ levels < 0.7 mmol/l) contributes to type 2 diabetes mellitus pathogenesis. Amongst other hypotheses, it has been suggested that Mg^2+^ deficiency affects insulin secretion. The aim of this study was, therefore, to investigate the acute effects of extracellular Mg^2+^ on glucose-stimulated insulin secretion in primary mouse islets of Langerhans and the rat insulinoma INS-1 cell line. Here we show that acute lowering of extracellular Mg^2+^ concentrations from 1.0 mM to 0.5 mM did not affect glucose-stimulated insulin secretion in islets or in insulin-secreting INS-1 cells. The expression of key genes in the insulin secretory pathway (e.g. *Gck*, *Abcc8)* was also unchanged in both experimental models. Knockdown of the most abundant Mg^2+^ channel *Trpm7* by siRNAs in INS-1 cells resulted in a 3-fold increase in insulin secretion at stimulatory glucose conditions compared to mock-transfected cells. Our data suggest that insulin secretion is not affected by acute lowering of extracellular Mg^2+^ concentrations.

## Introduction

Globally, the number of people that suffer from type-2 diabetes mellitus (T2DM) is steadily increasing, and the prevalence is predicted to pass the threshold of 500 million people by 2030 [1]. T2DM is characterized by impaired insulin secretion (i.e. insulin deficiency) and insulin sensitivity (i.e. insulin resistance), explaining the underlying pathophysiological mechanism for hyperglycemia (fasting serum blood glucose > 7 mmol/L) [2].

There is increasing evidence that hypomagnesemia (blood magnesium (Mg^2+^) < 0.7 mmol/L) is associated with T2DM pathogenesis and related complications [3]. Several epidemiological studies have shown that hypomagnesemia is higher in T2DM patients (14% - 48%) than in controls (2.5% - 15%) [3]. Indeed, a patient cohort of almost 400 T2DM patients showed that 30.6% suffered from hypomagnesemia with plasma Mg^2+^ levels below 0.7 mmol/L [4]. Furthermore, Mg^2+^ supplementation in T2DM patients improved insulin sensitivity and glucose metabolism [5–7].

Small scale clinical studies have demonstrated that insulin secretion may be lower in T2DM patients with hypomagnesemia [8, 9]. Supplementation of individuals without diabetes with MgCl_2_ significantly increased beta-cell function in a small randomized clinical trial [10]. However, experimental data concerning the role of Mg^2+^ in insulin secretion are conflicting, as both inhibitory and stimulatory effects have been reported in rodent models of hypomagnesemia. Isolated islets of rats on a Mg^2+^-deficient diet for 11 weeks showed a higher basal and glucose-stimulated insulin response [11]. In contrast, *Legrand et al* observed a reduced insulin response to glucose in rats on a Mg^2+^-deficient diet for 6 weeks compared to controls [12]. Interpretation of these contradictory results are further complicated since both short-term and long-term effects of Mg^2+^ may play a role.

In this study, we aimed to delineate the acute effects of extracellular Mg^2+^ concentrations on glucose-stimulated insulin secretion in isolated primary mouse islets of Langerhans and INS-1 cells under physiological and hyperglycemic conditions.

## Materials and methods

### Animal care

All procedures were conducted in compliance with the UK Animals Scientific Procedures Act (1986) and approved by the University of Oxford, Department of Physiology, Anatomy and Genetics Ethics Committee. Mice were housed in same-sex littermate groups of 2–8 animals, in a temperature and humidity-controlled room on a 12 hr light-dark cycle (lights on 7 am). Water and food (Special Diet Services, RM3) were available *ad libitum*. We used 10- to 16-week-old mice with a mixed (C3H, C57BL/6N, 129/sv) genetic background, as previously described [13]. Littermates consisting of wild-type mice, mice carrying Cre recombinase under the control of the rat insulin promoter (RIPII-Cre-ER mice), and mice carrying a transgene consisting of a floxed STOP codon upstream of a Kir6.2-V59M gene that was inserted into the ROSA locus (ROSA mice) (14), were used for insulin secretion experiments. The floxed Kir6.2 gene is not expressed due to the presence of the STOP codon. Likewise Cre-recombinase is not expressed as the gene is only activated when mice are injected with tamoxifen. We did not observe any differences in insulin secretion between these mice [14], or between these mice and wild-type mice on a pure C57bl/6N background.

### Pancreatic islet isolation

Mice were sacrificed by cervical dislocation, and pancreatic islets were isolated by collagenase digestion. In brief, the distal end of the common bile duct was tied near the anatomical transition to the duodenum, and the pancreas was distended by injecting HANKS (in mM: 137 NaCl, 2.5 CaCl_2_, 1.0 NaH_2_PO_4_, 5.6 KCl, 4.2 NaHCO_3_, 2.5 MgSO_4_ and 10 HEPES (pH 7.4 with NaOH)) containing Liberase (Roche, Mannheim, Germany) (0.33 mg/ml) into the common bile duct. After digestion at 37°C for 16 min, islets were washed in the same buffer, supplemented with 0.2% Bovine Serum Albumin (BSA) (Sigma, St. Louis, USA) and 0.05% D-glucose (Fisher Scientific, Leicestershire, UK). Islets were handpicked using a dissecting microscope into RPMI 1640 medium (Gibco, Darmstadt, Germany) containing 10% FBS (Gibco, Paisley, Scotland), 1% Penicillin-Streptomycin (Gibco, Paisley, Scotland) and 11 mM D-Glucose. They were either left for 24 hrs in this medium at 37°C in a humidified atmosphere with 5% CO_2_, or (as indicated) cultured for 24 hrs in home-made RPMI (S1 Table) with either normal (1.0 mM) or low (0.1 mM) extracellular magnesium. Islets from different mice were pooled in all experiments.

### Islet static insulin secretion

Groups of size-matched islets were handpicked and washed in Krebs-Ringer-bicarbonate (KRB) buffer: (in mM: 118.5 NaCl, 2.5 CaCl_2_, 1.2 KH_2_PO_4_, 4.7 KCl, 25 NaHCO_3_,1.2 MgCl_2_ and 10 HEPES (pH 7.4 with NaOH)), followed by a 0.5 hr pre-incubation in KRB without glucose (non-stimulant condition) at 37°C, 5% CO_2_ infusion. Static insulin secretion was assessed by challenging the islets with final concentrations of glucose and MgCl_2_ as indicated for 1 hr at 37°C, 5% CO_2_ infusion. The supernatant was collected to measure insulin secretion using a mouse insulin ELISA (Mercodia, Uppsala, Sweden). Islets were lysed in RIPA buffer (65 mM Tris-HCL, 150 mM NaCl, 5 mM EDTA, 1% NP-40, 0.5% Na-deoxycholate, 0.1% SDS, and 10% glycerol (pH 7.4 with HCl)) for 15 min on ice to extract total insulin content.

### Quantitative real time PCR

Islets were lysed directly after isolation or after culture for 24 hrs in home-made RPMI (S1 Table) with glucose and MgCl_2_, as indicated. Islets were lysed by Qiazol (Qiagen, Hilden, Germany) using the TissueLyser II, and chloroform (Sigma, St. Louis, USA) phase separation was used to extract total RNA. Purification was performed according to the manufacturer’s protocol, including an on-column DNase (Qiagen, Hilden, Germany) digestion step to eliminate genomic DNA. RNA was eluted in two steps using pre-heated RNA-free water and the concentration was checked using a Nano-drop ND-1000 spectrophotometer (Thermo Scientific, Wilmington, USA). Equal amounts of total RNA (0.1–1.5 μg) were reversed transcribed using the High Capacity cDNA Reverse Transcription kit (Applied Biosystems, Lithuania) according to the manufacturer’s protocol. The cDNA was subsequently used to determine the mRNA levels of *Trpm6*, *Trpm7*, *Slc41a1*, *Slc41a2*, *Slc41a3*, *Cnnm1*, *Cnnm2*, *Cnnm3*, *Cnnm4*, *Gck* and *Abcc8* in islets and *Trpm7*, *Cacna1c*, *Cacna1d*, *Ins1*, *Kcnj11*, *Abcc8*, and *Gck* in INS-1 cells using commercially available Taqman probes by the StepOnePlus Real-Time PCR System (Applied Biosystems) and normalized to *Actb* expression (Table 1). Data were analyzed using the Livak (2^-ΔΔCT^) method.

**Table 1:** Taqman gene expression assays used for RT-PCR analyses.

Target Gene	*Taqman Gene Expression Assays*
***Primary mouse islets of Langerhans***	
*Actb*	Mm00607939_s1
*Trpm6*	Mm00463112_m1
*Trpm7*	Mm00457998_m1
*Slc41a1*	Mm00715604_m1
*Slc41a2*	Mm01250930_m1
*Slc41a3*	Mm01182529_m1
*Cnnm1*	Mm00518996_m1
*Cnnm2*	Mm01205090_m1
*Cnnm3*	Mm01227346_m1
*Cnnm4*	Mm01227316_m1
*Gck*	Rn00561265_m1
*Abcc8*	Rn01476317_m1
***INS-1 cells***	
*Trpm7*	Rn01328216_m1
*Kcnj11*	Rn01764077_s1
*Abcc8*	Rn01476317_m1
*Cacna1c*	Rn00709287_m1
*Cacna1d*	Rn01453395_m1
*Ins1*	Rn02121433_g1
*Gck*	Rn00561265_m1

All Taqman Assays used for experiments in primary mouse islets of Langerhans (top) and INS1 cells (bottom) were commercially available (Applied Biosystems). Actb was used as control in all RT-PCRs.

### INS-1 cells

#### Culture

INS-1 832–13 cells were cultured in RPMI 1640 GlutaMax^TM^ (Gibco, Darmstadt, Germany) supplemented with 10% FBS, 50 μM β-mercaptoethanol (Sigma, St. Louis, USA), 1 mM sodium pyruvate (Sigma, St. Louis, USA), 1% HEPES (Gibco, Paisley, Scotland), and 1% pen/strep in a humidified atmosphere (37°C, 5% CO_2_). For mRNA expression analysis, INS-1 cells were seeded in 12-well plates (3 x 10^5^ cells per well) to reach confluence in 72 hrs. For the last 48 hrs, INS-1 cells were transfected with siTrpm7 (siGENOME SMARTpool siRNA, Dharmacon, Lafayette, USA) (final concentration 20 nM) and Lipofectamine**®** RNAiMAX reagent (Invitrogen, Carlsbad, USA) (1 ng/ml) in Opti-MEM (Gibco, Paisley, Scotland).

#### INS-1 static insulin secretion

INS-1 cells were cultured in 24-well plates (3 x 10^5^ cells per well) prior to the static secretion assay. INS-1 cells were washed in HBSS buffer (in mM: 114 NaCl, 4.7 KCl, 25.5 NaHCO_3_, 1.2 KH_2_PO_4_, 1.16 MgCl_2_, 20 HEPES, 2.5 CaCl_2_ (pH 7.4 with NaOH)), supplemented with 0.07% BSA. After pre-incubation for 2 hrs in HBSS without D-glucose (non-stimulant condition), INS-1 cells were challenged for 0.5 hr with final concentrations of D-glucose and MgCl_2_, as indicated. At the end of the incubation period, both the supernatant and the total insulin content (obtained by lysing cells in RIPA buffer) were collected, centrifuged for 5 min, at 14,000 rpm, 4°C, and directly stored at -20°C. Insulin secretion and total insulin content were determined using a mouse insulin ELISA kit.

#### Statistics

In all experiments, all data are expressed as the mean ± SEM. All statistical analyses were performed using GraphPad (Prism 7) software. Statistical comparisons were analyzed by an unpaired Student’s t-test or by a two-way ANOVA with a Tukey’s multiple comparison test. *p* < 0.05 was regarded as statistically significant.

## Results

### Acute lowering of Mg^2+^ does not affect insulin secretion in pancreatic β cells under normal- and hyperglycemic conditions

**Fig 1:**
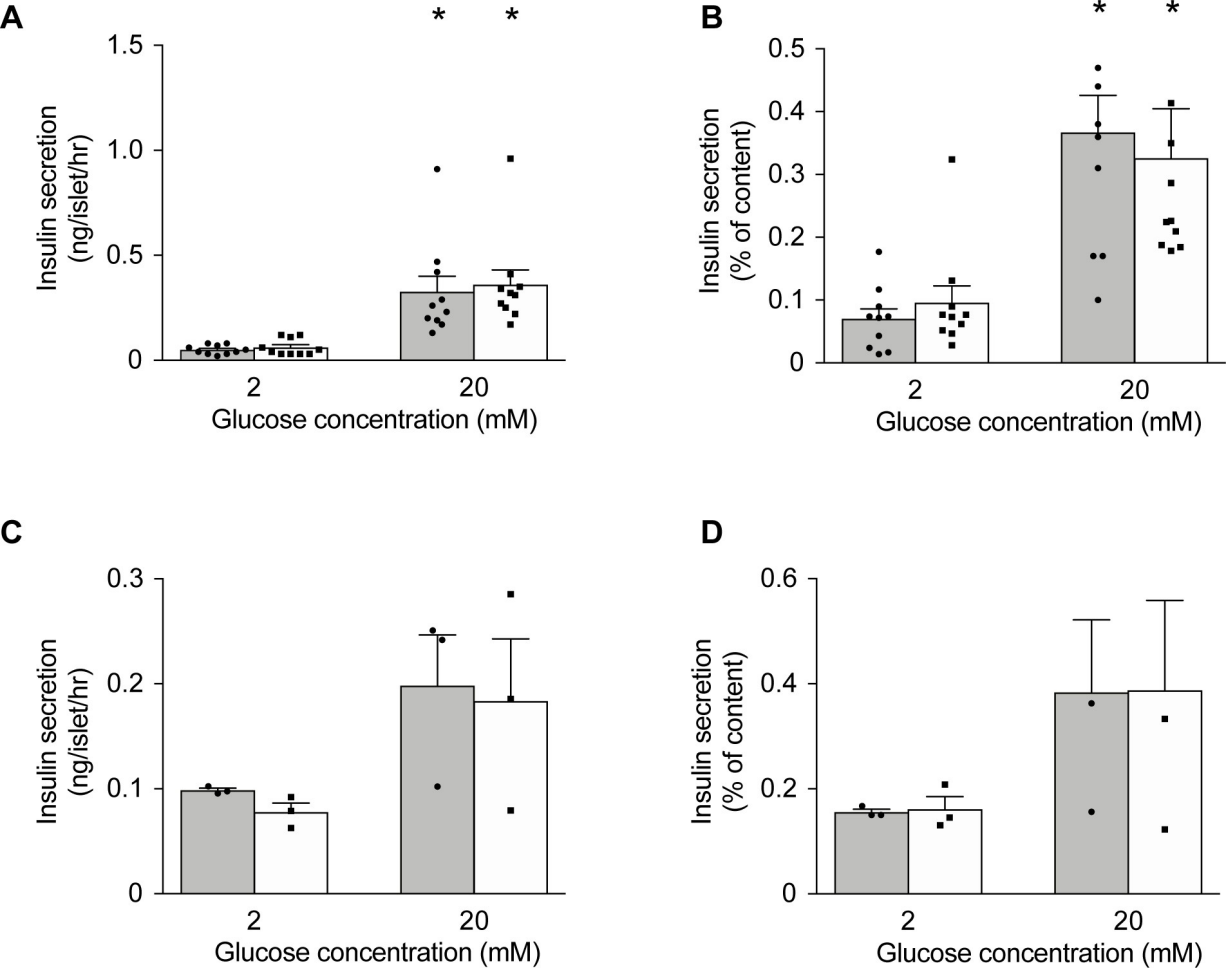
Acute lowering of Mg^2+^ does not affect insulin secretion in pancreatic β cells under normal- and hyperglycemic conditions. (**A-B**) Insulin secretion from isolated islets (n = 10 replicates, islets from 16 mice, 8 islets per replicate) challenged by 2 mM and 20 mM glucose at 0.5 mM Mg^2+^ (solid bar) and 1.0 mM Mg^2+^ (open bar) for 1 hr, after 24 hrs of culture at 11 mM glucose (1.2 mM Mg^2+^). (**C-D**) Insulin secretion from mouse pancreatic islets (n = 3, islets from 3 mice, 8 islets per replicate) stimulated by 2 mM and 20 mM glucose with 0.5 mM Mg^2+^ (solid bar) and 1.0 mM Mg^2+^ (open bar) for 1 hr after 24 hrs of culture at 25 mM glucose (1.2 mM Mg^2+^). Insulin secretion is presented as ng/islet/hr (**A, C**) and as normalized to total insulin content (**B, D**). Statistical significance was determined by two-way ANOVA. *, *p* < 0.05, 20 mM glucose vs. 2 mM glucose.

To investigate whether Mg^2+^ fulfils a functional role in pancreatic β cells in a normal and hyperglycemic environment, acute effects of extracellular Mg^2+^ on insulin secretion were examined in primary mouse islets of Langerhans (Fig 1). Insulin secretion at normal (1.0 mM) extracellular Mg^2+^ for 1hr increased 6-fold in response to elevation of glucose from 2 to 20 mM (Fig 1A) and increased 3-fold when expressed as a percentage of insulin content (Fig 1B). Reduction of extracellular Mg^2+^ from 1.0 to 0.5 mM for 1 hr had no effect on either basal insulin secretion or that stimulated by 20 mM glucose (Fig 1A and 1B). We next assessed the effects of low Mg^2+^ in combination with hyperglycemia. Culture at 25 mM glucose (1.2 mM Mg^2+^) for 24 hrs enhanced basal insulin secretion but was without effect on GSIS. Subsequent exposure to 0.5 or 1.0 mM Mg^2+^ for 1 hr did not alter basal insulin secretion or GSIS (Fig 1C and 1D). Likewise, increasing the time of culture to 48 hrs at normal (11 mM) or high (25 mM) glucose before acutely lowering extracellular Mg^2+^ did not alter the results (S1 Fig). Thus, acute exposure to low extracellular Mg^2+^ does not affect insulin secretion.

### Effects of prolonged exposure to low extracellular Mg^2+^ on insulin secretion

**Fig 2:**
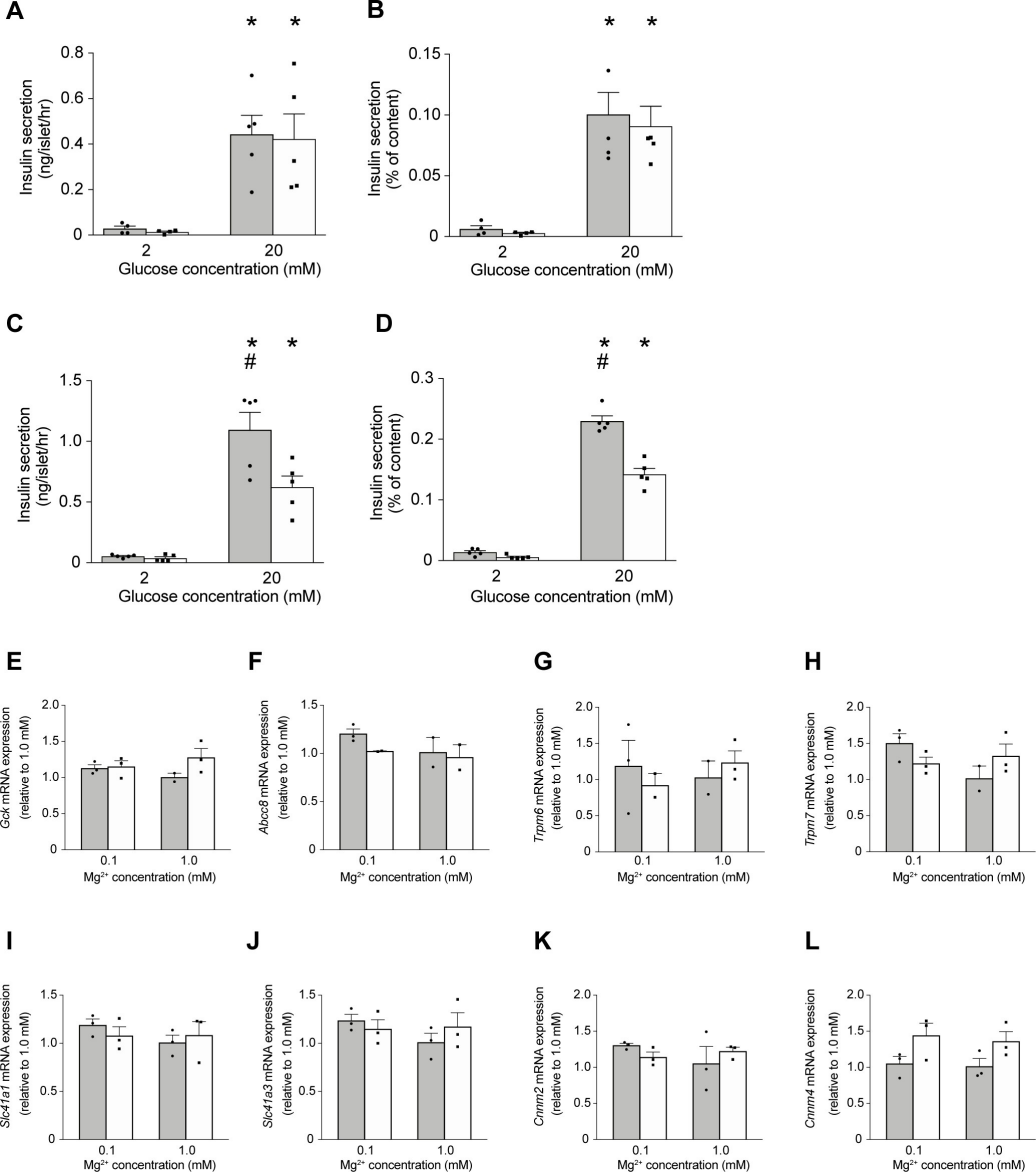
Effects of prolonged exposure to low extracellular Mg^2+^ on insulin secretion and gene expression. **(A-B)** Insulin secretion from isolated islets (n = 5 replicates, islets from 4 mice, 8 islets per replicate) challenged by 2 mM and 20 mM glucose at 0.1 mM Mg^2+^ (solid bar) and 1.0 mM Mg^2+^ (open bar) for 1 hr, after 24 hrs of culture at 11 mM glucose and either 0.1 mM or physiological (1.0 mM) extracellular Mg^2+^. (**C-D**) Insulin secretion from mouse pancreatic islets (n = 5 replicates, islets from 4 mice, 8 islets per replicate) stimulated by 2 mM and 20 mM glucose at 0.1 mM Mg^2+^ (solid bar) and 1.0 mM Mg^2+^ (open bar) for 1 hr after 24 hrs of culture at 25 mM glucose, and either 0.1 mM or 1.0 mM extracellular Mg^2+^. Insulin secretion is presented as ng/islet/hr (**A, C**) and as normalized to total insulin content (**B, D**). *, *p* < 0.05 (2 mM vs. 20 mM glucose); #, *p* < 0.05 (0.1 mM Mg^2+^, 20 mM glucose vs. 1.0 mM Mg^2+^, 20 mM glucose). Two-way ANOVA with a Tukey’s multiple comparison test. **(E-L)** The mRNA transcript levels of *Gck* (**E**), *Abcc8* (**F**), *Trpm6* (**G**), *Trpm7* (**H**), *Slc41a1* (**I**), *Slc41a3* (**J**), *Cnnm2* (**K**), *Cnnm4* (**L**) were measured in islets (n = 3 replicates, islets from 15 mice, 8 islets per replicate) after 24 hrs of culture at 11 mM glucose (solid bar), or 25 mM glucose (open bar) and either 0.1 mM Mg^2+^ or 1.0 mM Mg^2+^. mRNA expression levels were determined by quantitative RT-qPCR and normalized to *Actb* expression. Data are expressed relative to 1.0 mM Mg^2+^, 11 mM glucose.

To determine whether prolonged exposure to low extracellular Mg^2+^ affects GSIS, primary mouse islets of Langerhans were cultured for 24 hrs at either low (0.1 mM) or physiological (1.0 mM) extracellular Mg^2+^ and under both normal (11 mM) or hyperglycemic (25 mM) glucose concentrations. Insulin secretion was then measured in response to 2 and 20 mM glucose (Fig 2A–2D). In islets cultured at 11 mM glucose, elevation of glucose from 2 to 20 mM increased insulin secretion significantly at both low and normal Mg^2+^ (16-fold and 30-fold, respectively; Fig 2A and 2B). In islets cultured at 25 mM glucose, there was no difference in basal insulin secretion (2mM glucose) at low and normal Mg^2+^ (Fig 2C and 2D). However, insulin secretion at 20 mM glucose was significantly higher in islets exposed to 0.1 mM Mg^2+^ compared to 1.0 mM Mg^2+^ (Fig 2C and 2D). Thus, in islets cultured under hyperglycemic, low Mg^2+^ conditions, insulin secretion in response to 20 mM glucose is potentiated.

### Extracellular Mg^2+^ does not regulate mRNA levels of selected genes

The mRNA expression levels of selected genes were examined by RT-qPCR in islets cultured at 11 mM or 25 mM glucose and either 0.1 mM or 1.0 mM Mg^2+^. There were no significant effects on mRNA transcript levels of key Mg^2+^ channels and exchangers; *Trpm6*, *Trpm7*, *Slc41a1*, *Slc41a3*, *Cnnm2*, *and Cnnm4* (Fig 2G–2L). There were also no significant differences in mRNA expression levels of *Gck* and *Abcc8* between low Mg^2+^ and physiological Mg^2+^ concentrations and between normal and hyperglycemic glucose conditions (Fig 2E and 2F).

### Extracellular Mg^2+^ does not affect GSIS in INS-1 cells

**Fig 3:**
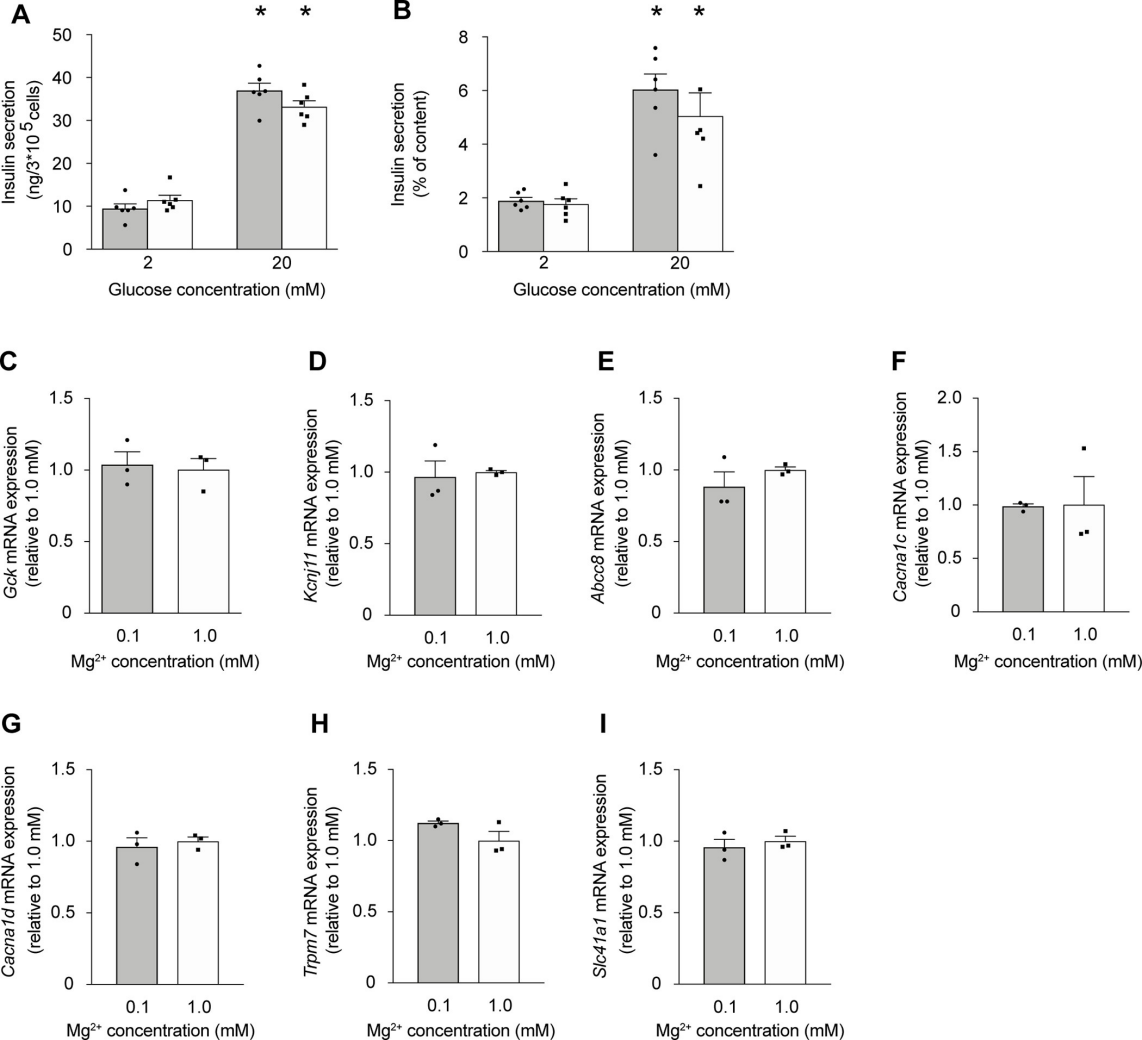
Extracellular Mg^2+^ does also not affect GSIS in INS-1 cells. (**A-B**) INS1 cells were cultured for 48 hrs at 11 mM glucose (1.2 mM Mg^2+^) followed by 24 hrs at 5 mM glucose (1.2 mM Mg^2+^) prior to the experiment. Insulin secretion from INS-1 cells stimulated by 2 mM and 20 mM glucose with 0.5 mM Mg^2+^ (solid bar) and 1.0 mM Mg^2+^ (open bar) for 0.5 hr (representative experiment from three independent experiments). Insulin secretion is presented as ng/3*10^5^ cells per hr (**A**) and normalized to total cell content (**B**). *, *p* < 0.05 (2mM vs. 20mM glucose) using two-way ANOVA. (**C-I**) The mRNA transcript levels of *Gck* (**C**), *Kcnj11* (**D**), *Abcc8* (**E**), *Cacna1c* (**F**), *Cacna1d* (**G**), *Trpm7* (**H**), and *Slc41a1* (**I**) were measured from INS-1 cells after 72 hrs of culture at standard glucose (11 mM) of which the last 48 hrs in low Mg^2+^ (0.1 mM) (solid bar) or physiological Mg^2+^ (1.0 mM) (open bar) culture conditions. mRNA expression levels were determined by quantitative RT-qPCR and normalized for *Actb* expression. Data are expressed relative to 1.0 mM Mg^2+^.

To verify our results from the experiments on primary mouse islets, we assessed the effects of low Mg^2+^ on GSIS in the rat insulinoma INS-1 cell line (Fig 3). Acute reduction of extracellular Mg^2+^ from 1.0 to 0.5 mM for 0.5 hr had no effect on either basal insulin secretion or that stimulated by 20 mM glucose (Fig 3A and 3B). Additionally, prolonged culture of 48 hrs under low Mg^2+^ conditions did not affect the transcript levels of *Gck*, *Kcnj11*, *Abcc8*, *Cacna1c*, and *Cacna1d* nor *Trpm7* and *Slc41a1* (Fig 3C–3I).

### *Trpm7* is the main Mg^2+^ channel in primary mouse islets

**Fig 4:**
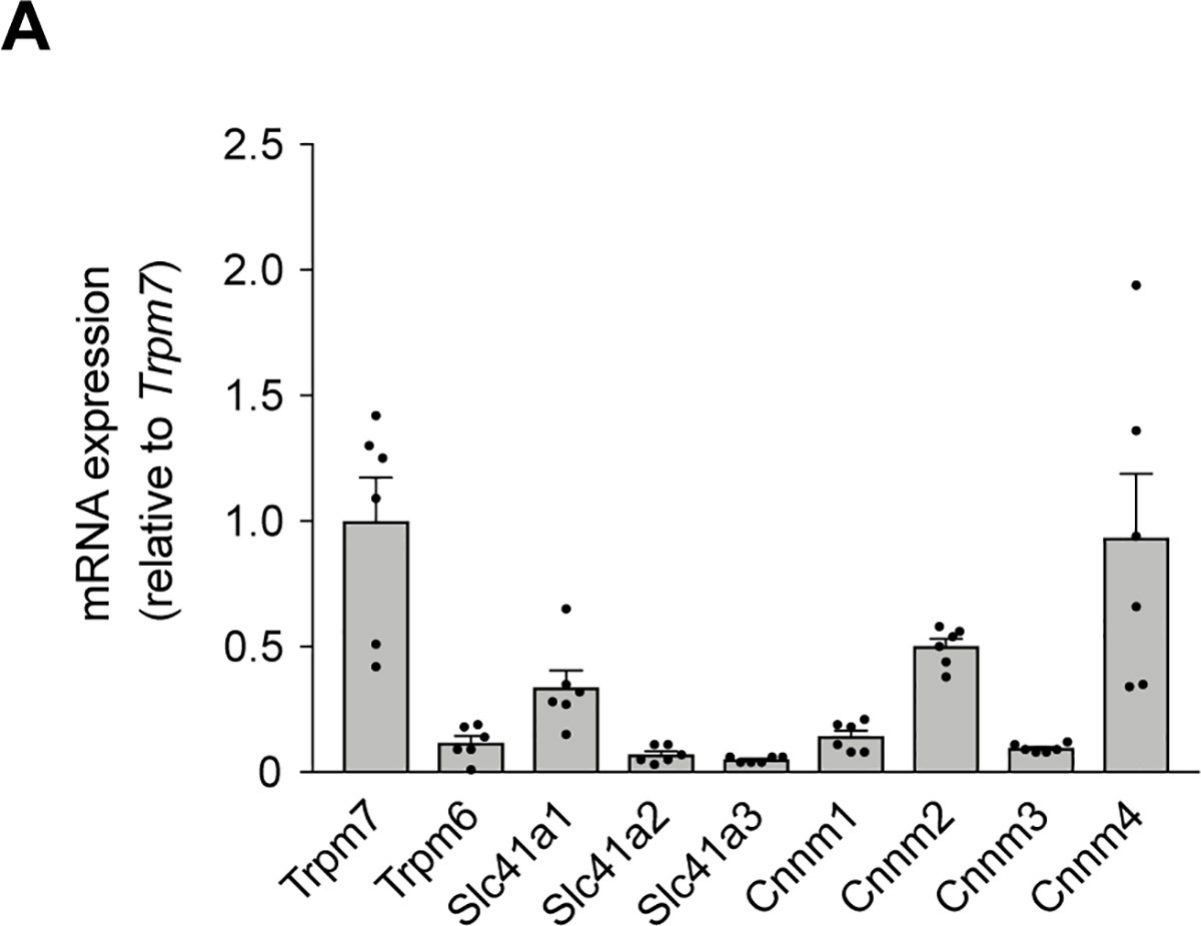
*Trpm7* is the main Mg^2+^ channel in primary mouse islets. (**A**) The mRNA transcript levels of *Trpm7*, *Trpm6*, *Slc41a1*, *Slc41a2*, *Slc41a3*, *Cnnm1*, *Cnnm2*, *Cnnm3*, and *Cnnm4* in islets (n = 6 replicates, islets from 6 mice). mRNA expression levels were determined by quantitative RT-qPCR and normalized to *Actb* expression. Data are expressed relative to *Trpm7* expression.

To understand which Mg^2+^ channels and transporters/exchangers play a pivotal role in pancreatic β cells, their mRNA expression levels were analyzed in primary mouse islets of Langerhans using RT-qPCR (Fig 4). RT-qPCR analysis demonstrated that the Mg^2+^ channel *Trpm7* and the Mg^2+^ exchanger *Cnnm4* were the highest expressed among those tested in primary mouse islets. Notably, *Trpm7* expression was 8-fold higher than its close homologue *Trpm6*, both of which are crucial for epithelial Mg^2+^ transport (Fig 4A).

### Knockdown of Trpm7 results in increased insulin secretion in INS-1 cells

**Fig 5:**
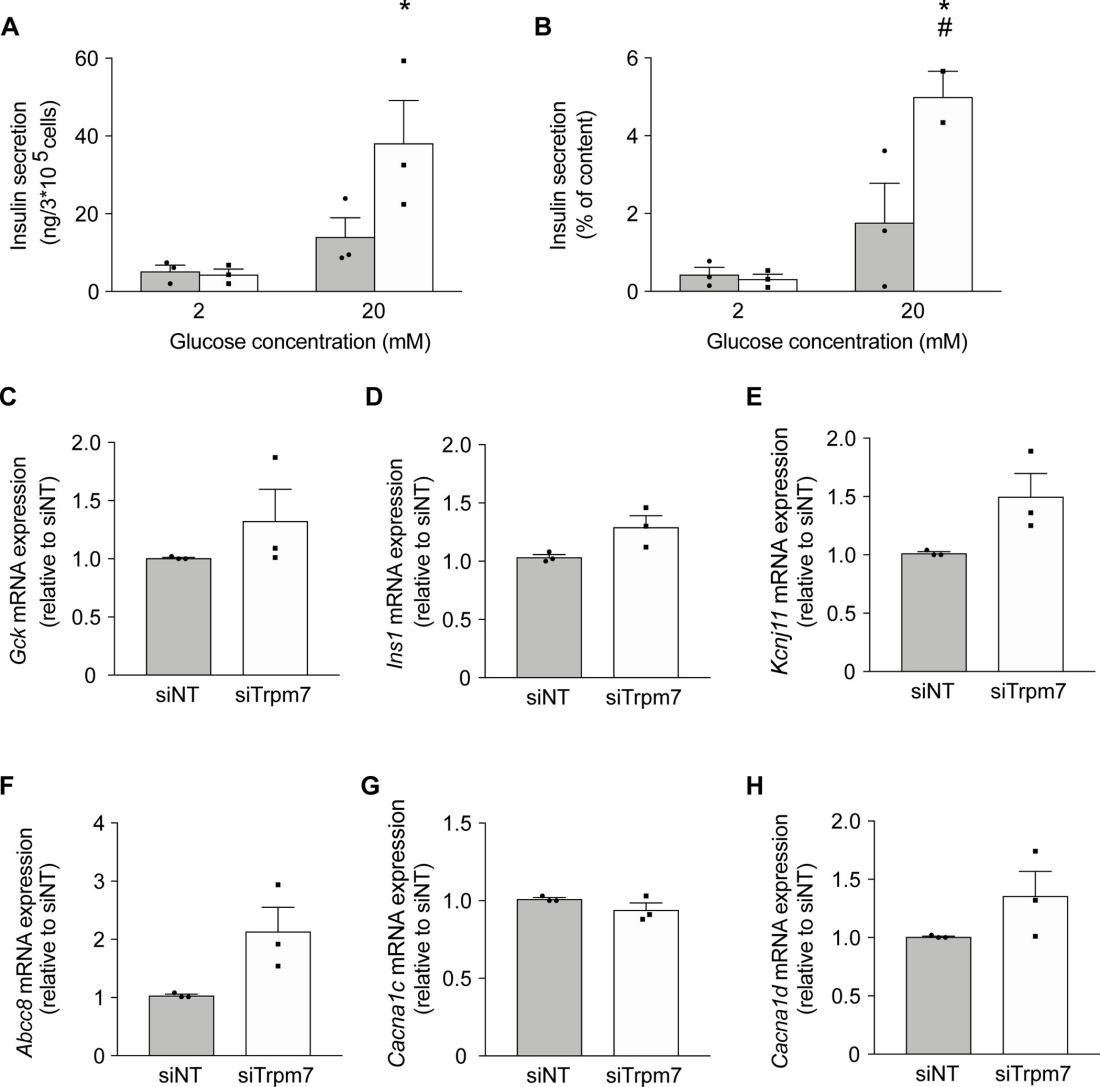
Knockdown of Trpm7 results in increased insulin secretion in INS-1 cells. **(A-B**) Insulin secretion from INS-1 cells (n = 3 experiments, 3 replicates each) stimulated by 2 mM and 20 mM glucose (1.0 mM Mg^2+^). Cells were cultured for 24 hrs at 11 mM glucose (1.2 mM Mg^2+^) followed by a further 48 hrs culture at 11 mM glucose (1.2 mM Mg^2+^) after transfection with siNON-targeting (siNT) (solid bar) or siTrpm7 (open bar). Insulin secretion is presented as ng/3*10^5^ cells per hour (**A**) and normalized to total cell content (**B**). *, *p* < 0.05 (2 mM vs. 20 mM glucose); #, *p* < 0.05 (siTrpm7, 20 mM glucose vs. siNT, 20 mM glucose). Two-way ANOVA. (**C-H**) The mRNA transcript levels of *Gck* (**C**), *Ins1* (*p* = 0.069) (**D**), *Kcnj11* (*p* = 0.069) (**E**), *Abcc8* (*p* = 0.058) (**F**), *Cacna1c* (**G**), *Cacna1d* (**H**) measured in INS-1 cells after 24 hrs of culture at 11 mM glucose (1.2 mM Mg^2+^) followed by a further 48 hrs culture at 11 mM glucose (1.2 mM Mg^2+^) after transfection with siNON-targeting (siNT) (solid bar) or siTrpm7 (open bar) (n = 3 experiments, 3 replicates each). mRNA expression levels were determined by quantitative RT-qPCR and normalized to *Actb* expression. Data are expressed relative to siNT levels.

Given the high expression levels of the Mg^2+^ channel *Trpm7*, we hypothesized that knockdown of *Trpm7* might reduce intracellular Mg^2+^ levels and thereby influence GSIS. *Trpm7* was knocked down using siRNA in INS-1 cells (Fig 5). *Trpm7* expression was 60% reduced by siRNA transfection (S2 Fig). Basal insulin secretion in *Trpm7* knockdown cells was similar to that in control cells. Secretion was significantly increased (by 16-fold) in response to 20 mM glucose in *Trpm7* knockdown cells (*p = 0*.*006*). At 20 mM glucose, there was significantly more secretion in *Trpm7* knockdown cells than control cells (Fig 5A and 5B). *Trpm7* knockdown did not affect the mRNA expression levels of key players in GSIS including *Gck*, *Ins1*, *Kcnj11*, *Abcc8*, *Cacna1c*, *and Cacna1d* (Fig 5C–5H).

## Discussion

This study demonstrates that reduced extracellular Mg^2+^ does not impair insulin secretion from pancreatic β-cells. This conclusion is based on the following results: *i)* Acute exposure to low extracellular Mg^2+^ has no effect on insulin secretion from either isolated mouse islets of Langerhans or INS-1 cells; *ii)* In islets cultured at 25 mM glucose, to mimic the hyperglycemic state found in T2DM, insulin secretion was actually enhanced by reducing extracellular Mg^2+^; *iii)* The mRNA expression levels of Mg^2+^ channels/transporters and key players in GSIS, in both islets and INS1 cells, were unaffected by extracellular Mg^2+^.

Previous experimental approaches have focused on the long-term effects of Mg^2+^ on insulin secretion in mouse and rat models. The results are conflicting. Reis *et al* showed a higher insulin response to basal and 8.3 mmol/L glucose concentrations in isolated islets of rats fed a Mg^2+^-deficient diet for 11 weeks [11]. In contrast, Legrand et al. observed a reduced insulin release [12]. The difficulty with long term Mg^2+^ deprivation is that both the extracellular and intracellular Mg^2+^ concentration may be reduced. In our approach, we aimed to separate the effects of extracellular Mg^2+^ and the effects of intracellular Mg^2+^. Interestingly, an acute reduction of extracellular Mg^2+^ did not affect insulin secretion in either primary islets or INS-1 cells. Similar findings were reported by an early study in perfused rat pancreas [15]. In contrast, knockdown of *Trpm7*, which is permeable to Mg^2+^_,_ significantly increased glucose-stimulated insulin release. This leads us speculate that changes in intracellular Mg^2+^ might modulate insulin release. Indeed, data from RIN m5F cells showed a stimulatory effect on insulin secretion when intracellular Mg^2+^ levels were reduced [16, 17].

Our results indicate that transient hypomagnesemia does not affect insulin secretion. However, patients with hypomagnesemia have chronic hypomagnesemia. Long term hypomagnesemia might reduce intracellular Mg^2+^ levels and thereby modify GSIS. Indeed, our results show that culturing islets for 24 hrs in a hyperglycemic environment under low Mg^2+^ conditions stimulated GSIS. This is in line with the negative correlation between magnesium levels and HOMA-B in a Canadian T2DM cohort [9].

Interestingly, our data show the importance of *Trpm7* in insulin secretion from pancreatic islets. *Trpm7* is the highest expressed Mg^2+^ channel. Although a major limitation of our study is the lack of measurement of intracellular Mg^2+^ levels, others have extensively reported that targeting *Trpm7* severely reduces intracellular Mg^2+^ levels [18–20]. Additionally, as TRPM7 can also function as a Ca^2+^ channel, we cannot exclude that TRPM7-mediated Ca^2+^ uptake explains the increased insulin secretion [21].

In recent years, several studies have shown that hypomagnesemia is a risk factor for progression of T2DM [3, 22]. Several hypotheses that could explain the mechanisms of this association have been proposed [23, 24]. Given the conflicting literature and our data showing that acute changes in extracellular Mg^2+^ do not inhibit insulin secretion in both pancreatic islets and INS-1 cells, we postulate that hypomagnesemia does not exert its effect primarily on insulin secretion. Based on recent large studies in animals and patients, other factors such as lipid metabolism may be more important in determining the interaction between hypomagnesemia and progression of type 2 diabetes [4, 25, 26].

In conclusion, we showed in different models that changes in extracellular Mg^2+^ do not impair glucose-stimulated insulin secretion. However, further research is needed to elucidate whether regulation of the intracellular Mg^2+^ concentration by *Trpm7* may influence insulin release.

## Supporting information

S1 Table Home-made RPMI medium. (DOCX)

S1 Fig GSIS following culture of isolated islets for 48 hrs. (**A-B**) Insulin secretion from isolated islets (n = 3 replicates, 9 mice, 8 islets per replicate) challenged with 2 mM and 20 mM glucose and 0.5 mM Mg^2+^ (solid bar) or 1.0 mM Mg^2+^ (open bar) for 1 hr after 48 hrs of culture at 11 mM glucose (1.2 mM Mg^2+^). (**C-D**) Insulin secretion from mouse pancreatic islets (n = 3 replicates, 9 mice, 8 islets per replicate) stimulated by 2 mM and 20 mM glucose with 0.5 mM Mg^2+^ (solid bar) and 1.0 mM Mg^2+^ (open bar) for 1 hr after 48 hrs of culture at 25 mM glucose (1.2 mM Mg^2+^). Insulin secretion is presented as ng/islet/hr (**A, C**) and normalized to total insulin content (**B, D).** *, *p* < 0.05 (2 mM vs. 20 mM glucose); Two-way ANOVA.
(DOCX)

S2 Fig Confirmation of *Trpm7* knockdown in INS-1 cells. (**A**) RT-qPCR of *Trpm7* mRNA in INS-1 cells (n = 3 experiments, 3 replicates each) following transfection with siNON-targeting (siNT) (solid bar) or siTrpm7 (open bar). mRNA expression levels were determined by quantitative RT-qPCR and normalized to *Actb* expression. Data are expressed relative to siNT. *, *p* < 0.05, Student’s t-test (two-tailed).
(DOCX)
