# Supplementary material for: Low extracellular magnesium does not impair glucose-stimulated insulin secretion
Source: PLoS One. 2019 Jun 4;14(6):e0217925. doi: 10.1371/journal.pone.0217925 (PMC6548430; doi:10.1371/journal.pone.0217925)
Supplement: S1 Table — (DOCX) [file pone.0217925.s001.docx]

**S1 Table. Home-made RPMI medium**

**Table 1.** Components (in mM) of home-made RPMI

| **Components** | **mM** |
| --- | --- |
| **Amino acids** | |
| Glycine | 0,133 |
| L-Arginine | 1,494 |
| L-Asparagine | 0,379 |
| L-Aspartic acid | 0,150 |
| L-Cystine 2HCl | 0,208 |
| L-Glutamic Acid | 0,136 |
| L-Glutamine | 2,055 |
| L-Histidine | 0,097 |
| L-Hydroxyproline | 0,153 |
| L-Isoleucine | 0,382 |
| L-Leucine | 0,382 |
| L-Lysine hydrochloride | 0,274 |
| L-Methionine | 0,101 |
| L-Phenylalanine | 0,091 |
| L-Proline | 0,174 |
| L-Serine | 0,286 |
| L-Threonine | 0,168 |
| L-Tryptophan | 0,025 |
| L-Tyrosine disodium salt dihydrate | 0,111 |
| L-Valine | 0,171 |
| **Vitamins** | |
| Biotin | 0,001 |
| Choline chloride | 0,021 |
| D-Calcium pantothenate | 0,006 |
| Folic Acid | 0,002 |
| Niacinamide | 0,008 |
| Para-Aminobenzoic Acid | 0,007 |
| Pyridoxine hydrochloride | 0,005 |
| Riboflavin | 0,006 |
| Thiamine hydrochloride | 0,003 |
| Vitamin B12 | 0,0000037 |
| i-Inositol | 0,194 |
| **Inorganic Salts** | |
| Calcium nitrate (Ca(NO3)2 4H2O) | 0,424 |
| Potassium Chloride (KCl) | 5,3 |
| Sodium Bicarbonate (NaHCO3) | 23,8 |
| Sodium Chloride (NaCl) | 103,4 |
| Sodium Phosphate dibasic (Na2HPO4-7H2O) | 5,6 |
| **Other components** | |
| Glutathione (reduced) | 0,003 |
| Phenol Red | 0,013 |
