# Supplementary material for: Low extracellular magnesium does not impair glucose-stimulated insulin secretion
Source: PLoS One. 2019 Jun 4;14(6):e0217925. doi: 10.1371/journal.pone.0217925 (PMC6548430; doi:10.1371/journal.pone.0217925)
Supplement: S1 Fig — (A-B) Insulin secretion from isolated islets (n = 3 replicates, 9 mice, 8 islets per replicate) challenged with 2 mM and 20 mM glucose and 0.5 mM Mg2+ (solid bar) or 1.0 mM Mg2+ (open bar) for 1 hr after 48 hrs of culture at 11 mM glucose (1.2 mM Mg2+). (C-D) Insulin secretion from mouse pancreatic islets (n = 3 replicates, 9 mice, 8 islets per replicate) stimulated by 2 mM and 20 mM glucose with 0.5 mM Mg2+ (solid bar) and 1.0 mM Mg2+ (open bar) for 1 hr after 48 hrs of culture at 25 mM glucose (1.2 mM Mg2+). Insulin secretion is presented as ng/islet/hr (A, C) and normalized to total insulin content (B, D). *, p < 0.05 (2 mM vs. 20 mM glucose); Two-way ANOVA. (DOCX) [file pone.0217925.s002.docx]

**S1 Fig. GSIS following culture of isolated islets for 48 hrs.**

**
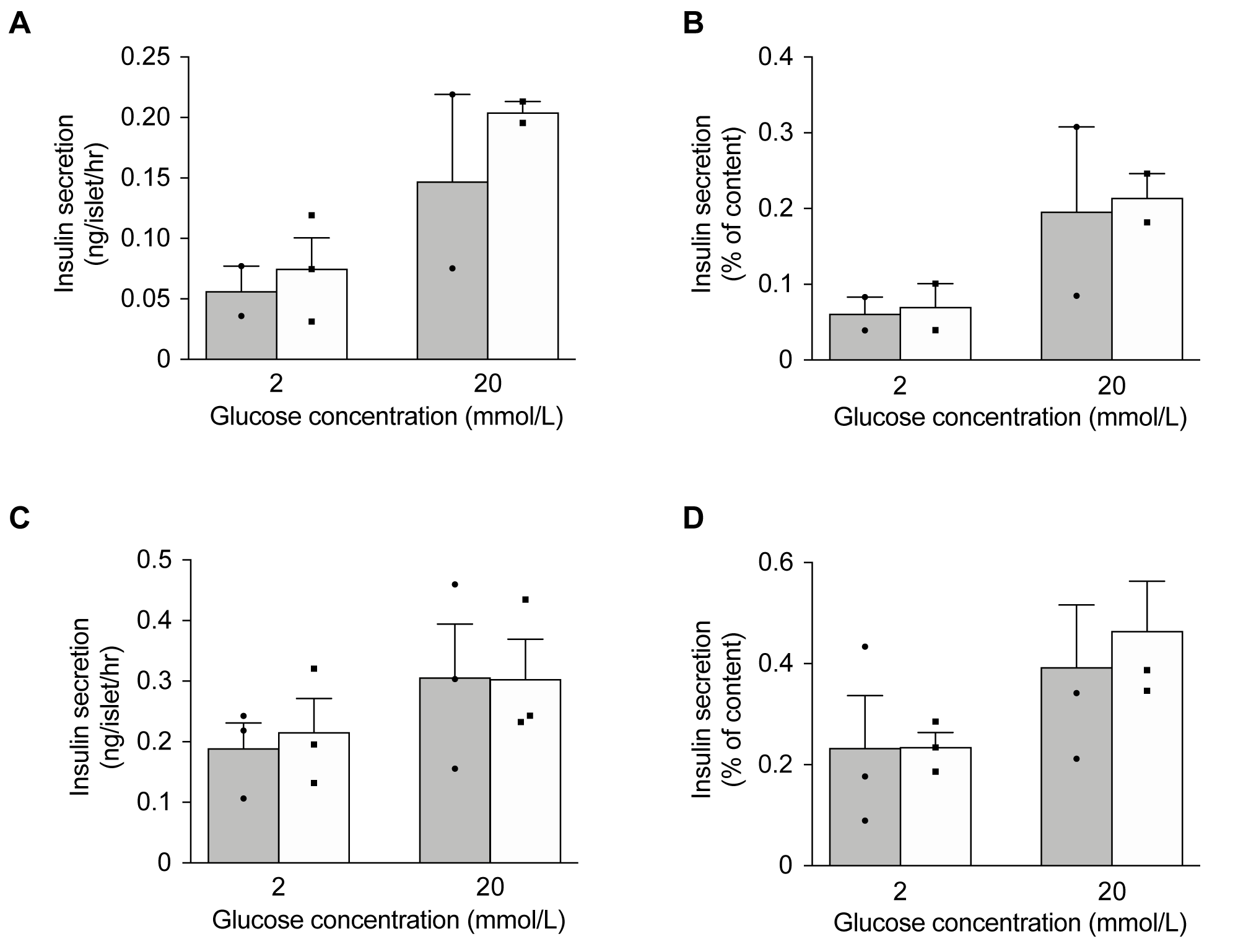
**

(**A-B**) Insulin secretion from isolated islets (n = 3 replicates, 9 mice, 8 islets per replicate) challenged with 2 mM and 20 mM glucose and 0.5 mM Mg^2+^ (solid bar) or 1.0 mM Mg^2+^ (open bar) for 1 hr after 48 hrs of culture at 11 mM glucose (1.2 mM Mg^2+^). (**C-D**) Insulin secretion from mouse pancreatic islets (n = 3 replicates, 9 mice, 8 islets per replicate) stimulated by 2 mM and 20 mM glucose with 0.5 mM Mg^2+^ (solid bar) and 1.0 mM Mg^2+^ (open bar) for 1 hr after 48 hrs of culture at 25 mM glucose (1.2 mM Mg^2+^). Insulin secretion is presented as ng/islet/hr (**A, C**) and normalized to total insulin content (**B, D).** ∗, *p* < 0.05 (2 mM vs. 20 mM glucose); Two-way ANOVA.
