# Supplementary material for: Low extracellular magnesium does not impair glucose-stimulated insulin secretion
Source: PLoS One. 2019 Jun 4;14(6):e0217925. doi: 10.1371/journal.pone.0217925 (PMC6548430; doi:10.1371/journal.pone.0217925)
Supplement: S2 Fig — (A) RT-qPCR of Trpm7 mRNA in INS-1 cells (n = 3 experiments, 3 replicates each) following transfection with siNON-targeting (siNT) (solid bar) or siTrpm7 (open bar). mRNA expression levels were determined by quantitative RT-qPCR and normalized to Actb expression. Data are expressed relative to siNT. *, p < 0.05, Student’s t-test (two-tailed). (DOCX) [file pone.0217925.s003.docx]

**S2 Fig. Confirmation of *Trpm7* knockdown in INS-1 cells.**


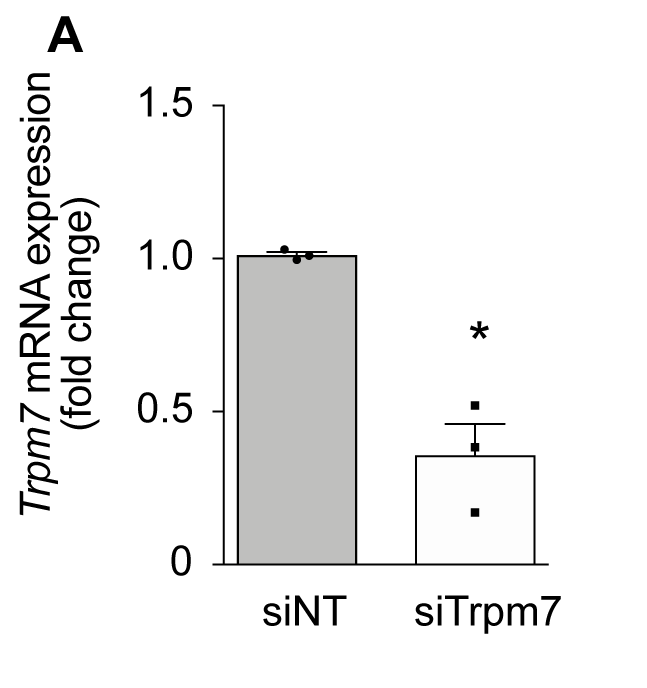


(**A**) RT-qPCR of *Trpm7* mRNA in INS-1 cells (n=3 experiments, 3 replicates each) following transfection with siNON-targeting (siNT) (solid bar) or siTrpm7 (open bar). mRNA expression levels were determined by quantitative RT-qPCR and normalized to *Actb* expression. Data are expressed relative to siNT. ∗, *p* < 0.05, Student’s t-test (two-tailed).
